# Inheritance of 2,4-dichlorophenoxyacetic acid (2,4-D) resistance in *Amaranthus palmeri*

**DOI:** 10.1038/s41598-022-25686-1

**Published:** 2022-12-17

**Authors:** Chandrima Shyam, Dallas E. Peterson, Amit J. Jhala, Mithila Jugulam

**Affiliations:** 1 Department of Agronomy, Kansas State University, 3703 Throckmorton Plant Sciences Center, 1712 Claflin Road, Manhattan, KS 66506 USA; 2 Department of Horticulture and Natural Resources, University of Nebraska-Lincoln, Lincoln, NE USA

**Keywords:** Plant sciences, Plant breeding

## Abstract

In this study, the inheritance of 2,4-D resistance in a multiple herbicide-resistant Palmer amaranth (KCTR) was investigated. Direct and reciprocal crosses were performed using 2,4-D-resistant KCTR and susceptible KSS plants to generate F_1_ progenies. 2,4-D dose–response assays were conducted to evaluate the response of progenies from each F_1_ family along with KCTR and KSS plants in controlled environmental growth chambers. Additionally, 2,4-D-resistant male and female plants from each of the F_1_ families were used in pairwise crosses to generate pseudo-F_2_ families. Segregation (resistance or susceptibility) of progenies from the F_2_ families in response to a discriminatory rate of 2,4-D (i.e., 560 g ae ha^−1^) was evaluated. Dose–response analysis of F_1_ progenies derived from direct and reciprocal crosses suggested that the 2,4-D resistance in KCTR is a nuclear trait. Chi-square analyses of F_2_ segregation data implied that 2,4-D resistance in KCTR is controlled by multiple gene(s). Overall, our data suggest that the 2,4-D resistance in KCTR Palmer amaranth is a nuclear inherited trait controlled by multiple genes. Such resistance can spread both via pollen or seed-mediated gene flow. In future, efforts will be directed towards identifying genes mediating 2,4-D resistance in KCTR population.

## Introduction

The introduction of herbicides in the 1940s revolutionized weed management in global agriculture. Herbicides are the primary tools used by growers to manage weeds in different cropping systems as well as in non-crop areas and home lawns. However, over-reliance on herbicides has resulted in the evolution of herbicide resistant weeds in many agriculturally important crops and cropping systems. There are currently 509 unique cases of herbicide resistance across 266 weed species worldwide, including 153 dicots and 113 monocots^1^. A critical aspect of the evolution of herbicide resistance in weeds is understanding how the resistance is inherited and spread^2^. Genetic factors, such as the degree of dominance of the trait and the number of alleles controlling the trait can impact how the trait can spread, which in turn will influence the weed management strategies^3^. For instance, herbicide resistance conferred by dominant gene(s) is expressed in both homozygous and heterozygous states, and therefore, can spread fast. In contrast, recessive traits can only express in a homozygous state and are slower to spread than dominant trait(s). If herbicide resistance is conferred by a single gene, it can spread faster compared to polygenic resistance traits, which require multiple recombination events during meiosis to accumulate multiple alleles (unless they are linked and inherit together). Knowledge of the inheritance of herbicide resistance in weeds can help understand the evolution of resistance under selection pressure^4^.

Herbicide resistance mechanisms in weeds/crops are broadly grouped into two categories, *i.e.*, target-site resistance (TSR) and non-target site resistance (NTSR). TSR mechanisms directly involve the modification to herbicide target and usually follow the Mendelian single gene inheritance model. In contrast, the NTSR mechanisms do not affect herbicide target, instead, alter the physiological processes involved in herbicide action^5^. Metabolism of herbicides, a common NTSR mechanism is reported to be conferred primarily by multiple genes; albeit in rare cases by a single gene^6–8^.

2,4-dichlorophenoxyacetic acid (2,4-D) is a synthetic auxinic herbicide (SAH) commonly used for controlling dicot weeds in cereal crops such as corn (*Zea mays* L.), wheat (*Triticum aestivum* L.), and sorghum (*Sorghum bicolor* L.). Despite being in use in agriculture for more than seven decades, the evolution of resistance to 2,4-D has been low. However, in the past decade, over-reliance on 2,4-D for controlling acetolactate synthase (ALS)- and 5-enol pyruvyl shikimate phosphate synthase (EPSPS)-inhibitor-resistant weeds, has led to an increase in weed resistance to SAHs. As of 2021, globally, 42 weeds are resistant to SAHs, amongst, 25 weeds have evolved resistance to 2,4-D^1^. This limit options for managing dicot weeds in cereal cropping systems. Moreover, increased adoption of commercialized 2,4-D-tolerant crop technology poses the risk of increased 2,4-D selection pressure, in several weeds^9^.

Inheritance of 2,4-D resistance has been studied in several economically important weeds around the globe, such as prickly lettuce (*Lactuca serriola* L.), wild radish (*Raphanus raphanistrum* L.), wild mustard (*Sinapis arvensis*), and oriental mustard (*Sisymbrium orientale* L.)^6,10–12^. So far, the inheritance pattern of SAHs such as 2,4-D resistance has varied depending on the resistance mechanism and the weed species involved. For instance, 2,4-D resistance in prickly lettuce was governed by a single co-dominant allele^12^. Similarly, in wild mustard (*Brassica kaber* L.) a single gene was found to mediate 2,4-D resistance^10^. However, polygenic inheritance was documented for MCPA resistance in hemp-nettle (*Galeopsis tetrahit* L.)^13^.

Palmer amaranth (*Amaranthus palmeri* S. Watson), a native of Mexico and the Southern United States, is one of the most common and troublesome weeds in the United States^14^. So far, Palmer amaranth has evolved resistance to 8 herbicide sites of action (SOAs) ^1^. The reproductive biology of Palmer amaranth plays a crucial role in its ability to evolve resistance to herbicides. For instance, Palmer amaranth is a dioecious plant, making cross-pollination a necessity to produce seeds^15,16^. Female Palmer amaranth plants are characterized by high fecundity resulting in the production of abundant seeds per plant^17,18^.

We documented a multiple resistant population of Palmer amaranth (Kansas Conservation Tillage resistant; KCTR) from Kansas which has evolved resistance to six herbicides, including 2,4-D^19,20^. Furthermore, recently we reported enhanced metabolism of 2,4-D in KCTR compared to KSS and MSS indicating metabolic resistance, even though research on possible co-existence of TSR is warranted^19^. Although resistance to 2,4-D in Palmer amaranth has been documented in two populations in Kansas^19–21^, the information on the inheritance of 2,4-D resistance is lacking. This study was conducted to investigate the inheritance of 2,4-D resistance in KCTR Palmer amaranth. The specific objectives were: i) generate F_1_ progenies and evaluate their response to 2,4-D application, and ii) generate F_2_ progenies and determine if 2,4-D resistance in KCTR is conferred by either single or multiple genes.

## Results

### 2,4-D dose–response with KCTR, KSS, and F1 progenies

**Figure 1:**
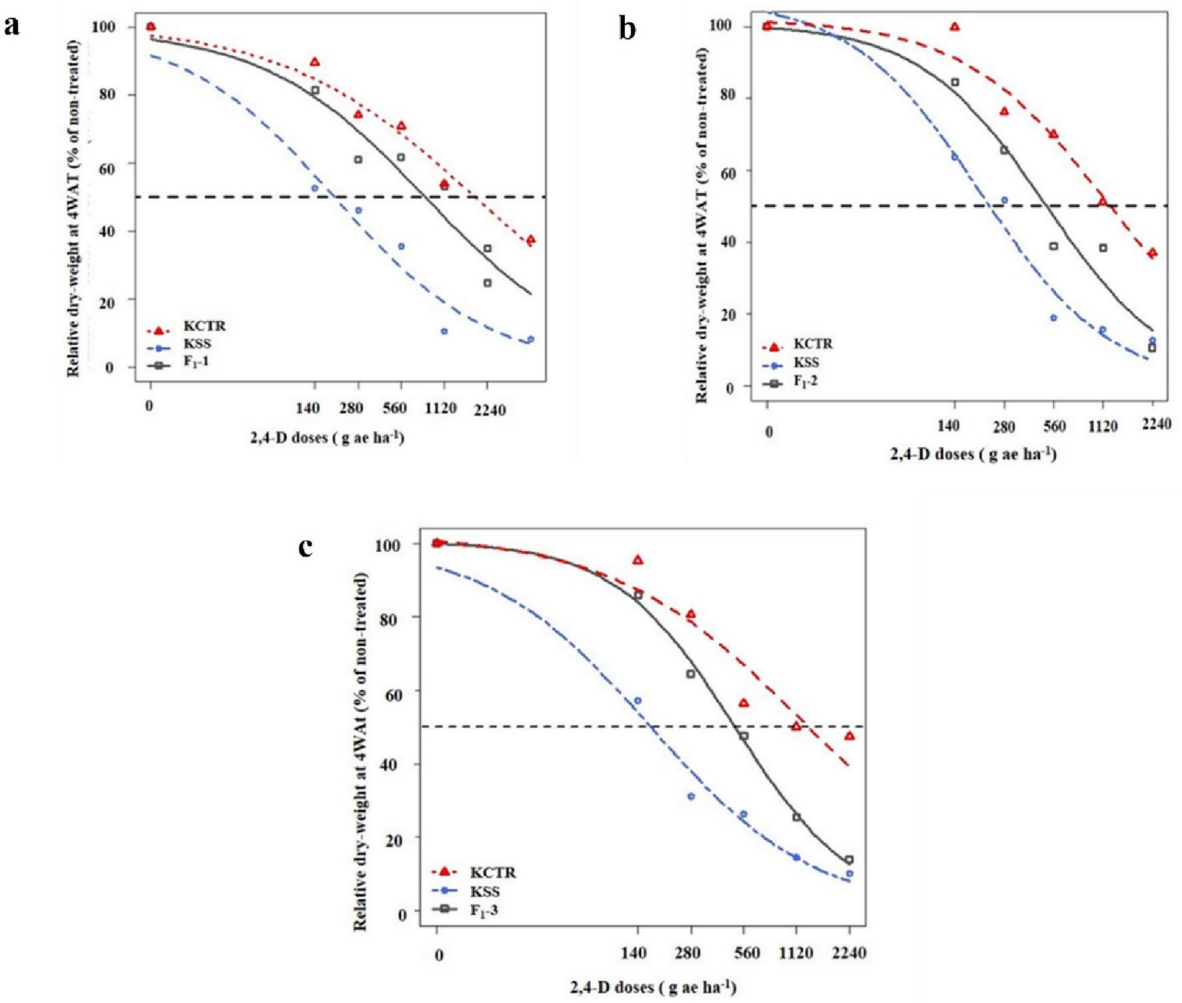
Dose–response curves representing the level of 2,4-D resistance in (**a**) F_1_-1, (**b**) F_1_-2, and (**c**) F_1_-3 progenies compared to parental 2,4-D susceptible (KSS), and 2,4-D resistant (KCTR) Palmer amaranth. Relative shoot dry weight (% of non-treated) of seedlings were analyzed using the three-parameter log-logistic regression model (Eq. 2) at four weeks after treatment. Points in each plot indicate experimental response of the F_1_ plants to respective rates of 2,4-D. Dotted arrow at the center of the plots represents the 50% of relative dry weight.

**Figure 2:**
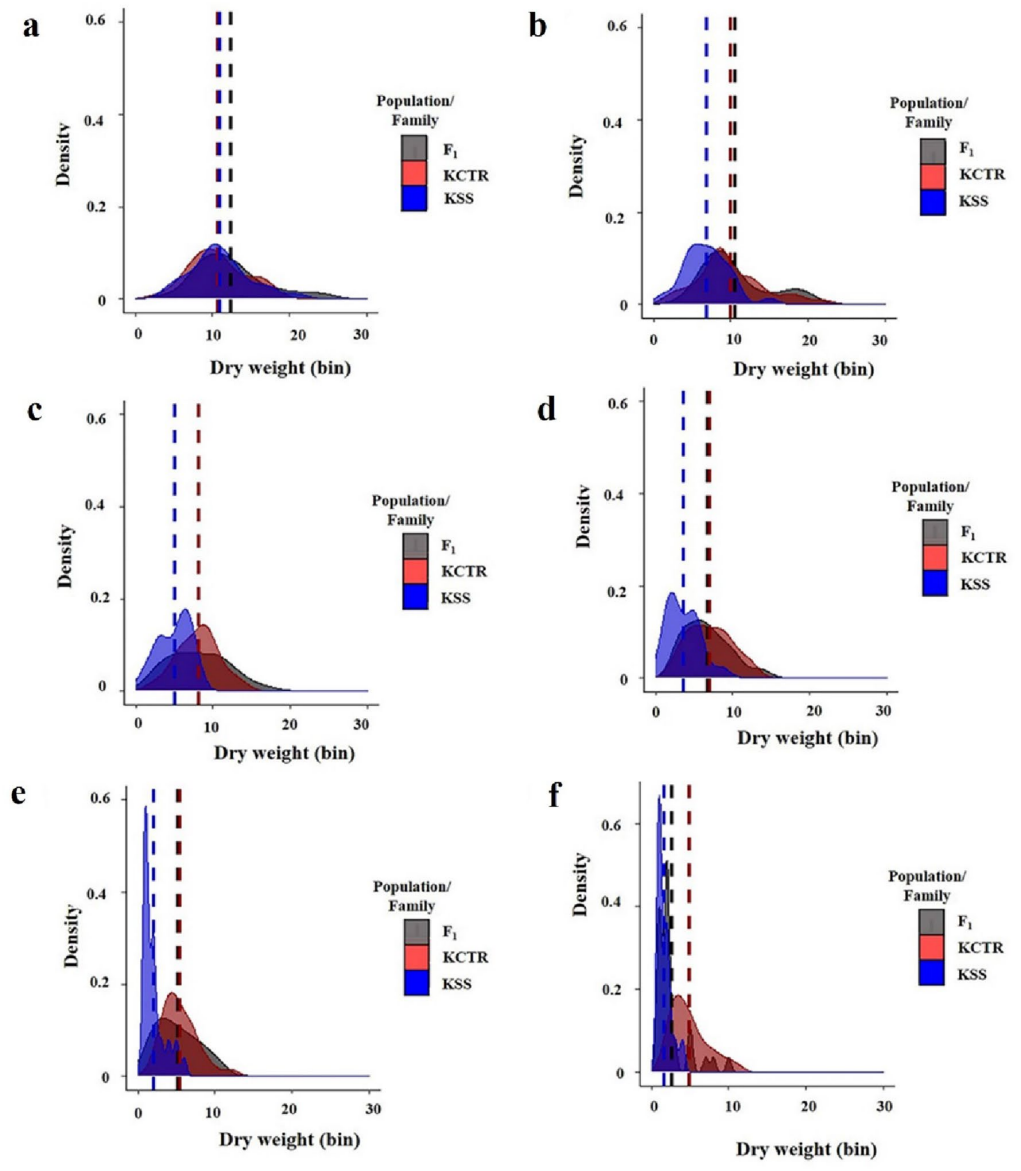
Cumulative dry weight distribution of 2,4-D susceptible (KSS), resistant (KCTR), Palmer amaranth and F_1_ progenies in response to 2,4-D at (**a**) 0 (non-treated) (**b**) 140, (**c**) 280, (**d**) 560 (field recommended dose), (**e**) 1120, and (**f**) 2240 g ae ha^−1^ via density plots. Dry weight of samples was converted into bins and plotted in the X-axis. Y-axis was plotted by calculating density which is the proportion of total plants in each bin. Dotted lines represent mean dry weight of KSS, KCTR, and F_1_ plants.

**Figure 3:**
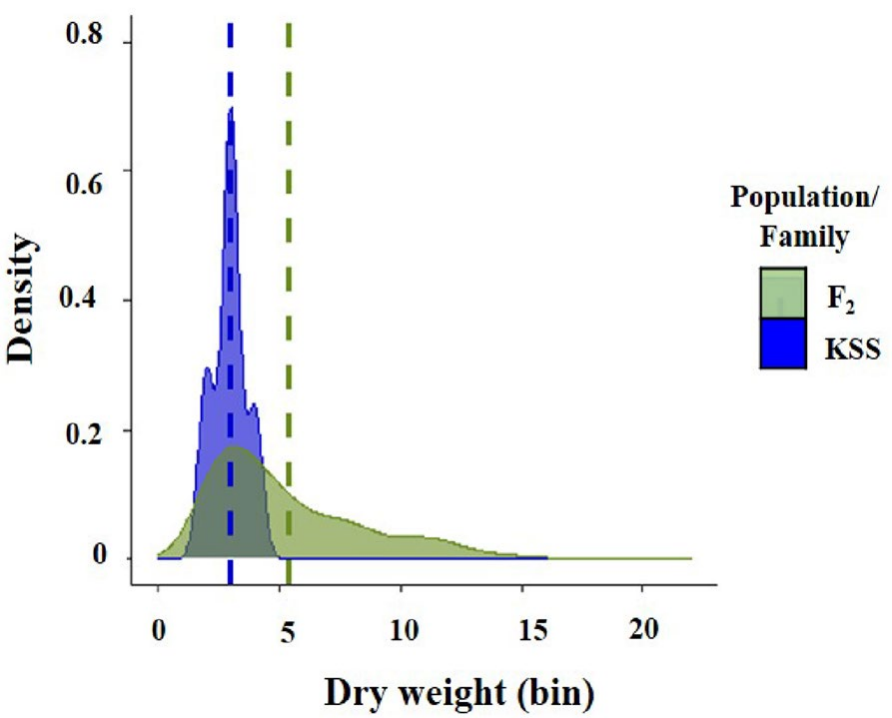
Cumulative dry weight data distribution of 2,4-D susceptible (KSS; n = 21) and F_2_ progenies (n = 332) in response to 2,4-D at 560 g ae ha^−1^ (field recommended dose) via density plot. Dry weight of samples was converted into bins and plotted on the X-axis. Y-axis was plotted by calculating density which is the proportion of total plants in each bin. Dotted lines represent mean dry weight of 2,4-D susceptible (KSS) and F_2_ plants.

Response of progenies from F_1_ families and parental Palmer amaranth populations, KCTR and KSS to 2,4-D was assessed via dose-responses experiments. Our data indicates that 90% of KCTR and 15% of KSS plants survived the discriminatory dose (1120 g ae ha^−1^) of 2,4-D. The KSS plants which survived 2,4-D treatment, showed severe epinasty (downward curling of plant parts, a typical symptom of auxinic herbicide injury) and stayed stunted and did not show any new growth up to 4WAT. Nonetheless, several KCTR Palmer amaranth plants survived even the highest dose used in the study (2240 g ae ha^−1^). Compared to that, 87% of F_1_-1, 92% of F_1_-2, and 89% of F_1_-3 survived application of 2,4-D at 1120 g ae ha^−1^. The F_1_ plants in all the three families, generated by both direct or reciprocal crosses survived 2,4-D application, implying that the resistance to 2,4-D in KCTR Palmer amaranth is a nuclear trait. In the F_1_-1 dose–response experiments, the amount of 2,4-D required to cause 50% reduction in growth (GR_50_) of KCTR, F_1_-1 and KSS were 1800, 837, and 194 g ae ha^−1^, respectively (Table 1; Fig. 1), suggesting that the KCTR and F_1_-1 are 9- and fourfold resistant to 2,4-D compared to KSS (Table 1). Similar results were obtained from F_1_-2 and F_1_-3 dose–response experiments (Tables 2, 3; Figs. 2, 3). The GR_50_ of KCTR, KSS, and F_1_-2 plants were 1188, 501, and 200 g ae ha^-1^, respectively (Table 2; Fig. 2), indicating that the KCTR and F_1_-2 plants were 6- and threefold resistant, respectively, to 2,4-D treatment compared to KSS. Likewise, the GR_50_ of KCTR, KSS, and F_1_-3 were 1317, 500, and 167 g ae ha^−1^, respectively (Table 3; Fig. 3), with 7 and 3 RI compared to KSS (Table 3). P-values from the t-test for each F_1_ family are presented in supplementary material (Table S1). Overall, the results of 2,4-D dose–response experiments suggest an intermediate response of F_1_ progenies compared to KCTR and KSS plants.

**Table 1:** Regression parameters describing the response of F_1_-1 progenies along with 2,4-D susceptible (KSS), resistant (KCTR) Palmer amaranth to 2, 4-D under growth chamber conditions (Eq. 2).

F_1_-family/population	b (SE)	d (SE)	e (SE)	RI
F_1_-1	0.76 (0.12)	99.60 (4.57)	837.31 (162.98)	4.3
KCTR	0.65 (0.13)	100.74 (5.32)	1800 (566.45)	9.3
KSS	0.81 (0.16)	99.68 (5.52)	194 (46.96)	–

**Table 2:** Regression parameters describing the response of F_1_-2 progenies along with 2,4-D susceptible (KSS), resistant (KCTR) Palmer amaranth to 2, 4-D under growth chamber conditions (Eq. 2).

F_1_-family/population	b (SE)	d (SE)	e (SE)	RI
F_1_-2	1.13 (0.23)	100.80 (6.71)	501.18 (106.62)	2.5
KCTR	0.99 (0.23)	102.11 (6.69)	1188.02 (288.70)	5.9
KSS	1.09 (0.29)	107.87 (8.83)	200.02 (53.59)	–

**Table 3:** Regression parameters describing the response of F_1_-3 progenies along with 2,4-D susceptible (KSS), resistant (KCTR) Palmer amaranth to 2, 4-D under growth chamber conditions (Eq. 2).

F_1_-family/population	b (SE)	d (SE)	e (SE)	RI
F_1_-3	1.27 (0.24)	100.61 (6.07)	500.2 (90.43)	3.0
KCTR	0.80 (0.17)	102.74 (5.61)	1316.97 (343.25)	7.9
KSS	0.93 (0.22)	100.31 (6.61)	166.50 (43.50)	–

Further, cumulative dry weight data from the F_1_ dose–response assays were plotted for KSS, KCTR, and F_1_ plants at each 2,4-D dose (Fig. 2). The spread and distribution of dry weight of non-treated (NT; 0 g ae ha^−1^) KSS, KCTR, and the F_1_ plants were similar (Fig. 2a). At doses up to 2× (1120 g ae ha^−1^), F_1_ and KCTR plants had visually similar distribution (Fig. 2b–e), however, at 4× dose (4×; 2240 g ae ha^−1^), KCTR plants had a wider spread than F_1_ plants (Fig. 2f), indicating higher level of resistance compared to F_1_ progenies.

### Segregation (2,4-D resistance or susceptibility) of F_2_ progenies

**Figure 4:**
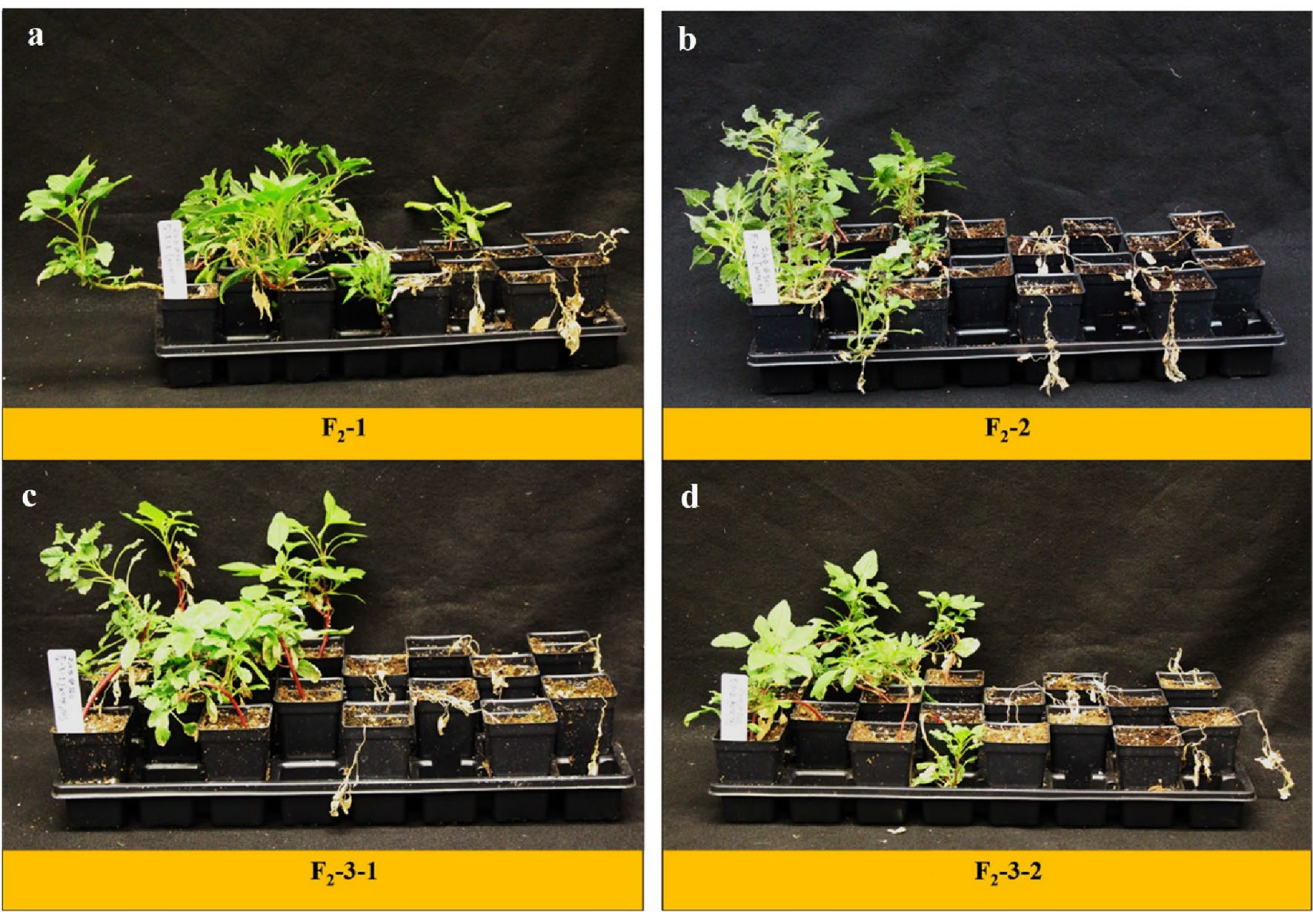
Diverse phenotype of F_2_ progenies from family (**a**) F_2_-1, (**b**) F_2_-2, (**c**) F2-3-1, and (**d**) F_2_-3-2 in response to 2,4-D at 560 g ae ha^−1^ (field recommended dose) at 4 weeks after treatment.

Response of progenies from F_2_ families and KSS population (as control) were evaluated by treating with 2,4-D at the field recommended dose of 560 g ae ha^−1^. Unfortunately, as mentioned in the methods, the KCTR plants were not used in this experiment along with KSS Palmer amaranth due to lack of enough seed and poor germination at the time of conducting this experiment. As expected, upon treatment with 560 g ae ha^−1^ of 2,4-D, all KSS plants (n =  ~ 50), either completely died or had dried leaves and stunted growth at 4 WAT (Table 4). Interestingly, the percent survival of F_2_ progenies highly varied among families. The phenotypes of F_2_ progenies ranging from high resistance (plants showing low epinasty and high dry weight accumulation to very low-level resistance (plants showing high epinasty and low dry weight accumulation) were observed (Fig. 4). In the first run of the segregation assay, all F_2_ families deviated from the expected 3:1 (resistance:susceptibility) segregation ratio (Table 4). In the second run of the segregation assay, except for the F_2_-1 family, all other families did not follow the 3:1 (resistance and susceptible) segregation ratio (Table 4). Overall, when combined (run 1 and 2; Table 4) all F_2_-families failed to segregate as 3:1 (resistance and susceptible) ratio, indicating that more than one gene(s) is involved in mediating 2,4-D resistance in KCTR.

**Table 4:** Chi-square test for goodness of fit of the observed segregation of plants as resistance or susceptible (Eq. 4) to 2,4-D to the expected frequency for a single-locus model in pseudo-F_2_ families of Palmer amaranth.

Run	F_2_ family/population	Plants treated	Observed survival		Expected survival		p-value
			Alive	Dead	Alive	Dead	
1	F_2_-1	72	30	42	54	18	< 0.00001
	F_2_-2	101	56	45	75.75	25.25	< 0.00001
	F_2_-3-1	74	46	28	55.5	18.5	0.01076
	F_2_-3-2	53	25	28	39.75	13.25	< 0.00001
	KSS	31	0	31	–	–	–
	Total	300	157	143	225	75	< 0.00001
2	F_2_-1	92	63	29	69	23	0.14856
	F_2_-2	20	9	11	15	5	0.00195
	F_2_-3-1	124	79	45	93	31	0.00369
	F_2_-3-2	96	46	50	72	24	< 0.00001
	KSS	20	0	21	–	–	–
	Total	332	197	135	249	83	< 0.00001
1 & 2 combined	F_2_-1	164	93	71	123	41	< 0.00001
	F_2_-2	121	65	56	90.75	30.25	< 0.00001
	F_2_-3-1	198	125	73	148.5	49.5	0.00011
	F_2_-3-2	149	71	78	111.75	37.25	< 0.00001
	KSS	51	0	51	–	–	–
	Total	632	354	278	474	158	< 0.00001

The distribution of shoot dry weight per plant (g) of the F_2_ progenies (n = 332) and KSS plants (n = 21) was illustrated through a density plot (Fig. 3). The distribution showed a wide spread of dry weight bins for F_2_ progenies representing high plant to plant variation in response to 2,4-D treatment (Fig. 3).

## Discussion

The evolution of 2,4-D resistance in Palmer amaranth has been reported in two populations, from Kansas, USA, including, KCTR^19–21^. KCTR has been documented to have evolved predominantly metabolic resistance to at least five herbicide SOAs including ALS-, photosystem (PS) II-, protoporphyrinogen oxidase-, 4-hydroxyphenylpyruvate dioxygenase- inhibitors and SAHs^20^. Information on the genetic basis of 2,4-D resistance in Palmer amaranth is lacking. Therefore, in the current study, we investigated the inheritance of 2,4-D resistance in KCTR.

In the dose–response assays, KCTR plants, as expected showed a high level of 2,4-D resistance (6–ninefold) compared to KSS plants (Fig. 1a–c; Tables 1, 2, 3). Progenies generated by both direct and reciprocal crosses were resistant to 2,4-D, indicating that the 2,4-D resistance in KCTR is a nuclear trait (Fig. 1; Tables 1, 2, 3). In general, resistance to majority of herbicides is a nuclear trait. One exception is the TSR to PS II-inhibitors in weeds, which is inherited maternally/cytoplasmically^22^. Nuclear inheritance of 2,4-D inheritance has been reported in several weeds, including wild radish, wild mustard, oriental mustard, and prickly lettuce^6,10–12^. Nuclear traits are transferred by both seed and pollen of resistant plants. Previously, pollen-mediated intra- and interspecific transfer of TSR and NTSR traits have been documented in *Amaranthus *sp.^15,16,23–26^. Therefore, there is a potential of inter-and intra-specific transfer of 2,4-D resistance via pollen-mediated gene flow.

The results of F_1_ dose–response assay showed an intermediate level of 2,4-D resistance in all F_1_ families compared to the parental resistant (KCTR) and susceptible (KSS) plants (Figs. 1, 2; Tables 1, 2, 3). Analyses of data from both dose–response studies and distribution plotting of cumulative biomass of KCTR, KSS and F_1_ progenies (Figs. 1, 2; Tables 1, 2, 3) confirmed that 2,4-D resistance in KCTR Palmer amaranth is a nuclear trait and transferred to progenies generated from both direct and reciprocal crosses. Most cases of herbicide resistance, including TSR or NTSR, have been reported to be controlled by either completely or incompletely dominant genes^6,11,26,27^. The 2,4-D resistance in wild radish was found to be incompletely dominant ^6^. In contrast, inheritance of 2,4-D resistance in wild radish and dicamba resistance in kochia (*Bassia scoparia* L.) were found to be mediated by highly dominant alleles^6,28,29^. Both dominant and incompletely dominant traits can be expressed in homo- or heterozygosity, therefore, the spread of 2,4-D resistance will be faster than a recessive trait.

Segregation of F_2_ progenies in KCTR Palmer amaranth at the field recommended dose of 2,4-D (560 g ae ha^−1^) did not follow the Mendelian 3:1 (resistance: susceptible) ratio (expected for a single gene inheritance) (Table 4). This indicates that the 2,4-D resistance in KCTR Palmer amaranth is a polygenic trait. Furthermore, the chi-square goodness of fit test data suggests variability in the phenotypes among the F_2_ resistant progenies, which cannot be explained by a single major gene-mediated resistance (Fig. 3). Although majority of TSR is controlled by a single gene^30,31^, the inheritance of NTSR was reported to be conferred by both single and multiple genes^7,8,25–27^. For instance, monogenic metabolic resistance to chlorsulfuron and atrazine was documented in common waterhemp (*Amaranthus tuberculatus* Moq. Sauer.), Palmer amaranth, and sorghum^7,20,26,27,32^. Polygenic inheritance was reported for mesotrione resistance in common waterhemp and Palmer amaranth^7,26,27,32^. Similar to our findings, MCPA resistance in hemp-nettle was found to be polygenic^13^. On the other hand, single-gene inherited 2,4-D resistance was reported in wild mustard, oriental mustard, prickly lettuce^10–12^. Traits inherited by multiple genes spread slower than those with single alleles.

Our previous work indicates that KCTR Palmer amaranth evolved metabolic resistance, possibly mediated by cytochrome P450 activity^19,20^. In other 2,4-D, resistant weed species, such as common waterhemp metabolic resistance mediated by P450 activity has been reported^33,34^. Increased expression of a P450 cluster (*P450-81E8*) was recently reported in 2,4-D-resistant common waterhemp populations from both Nebraska and Illinois^35^. We have reported increased expression of this gene in KCTR plants with metabolic resistance to tembotrione^36^. Therefore, there is a need to check expression levels of this gene in 2,4-D resistant KCTR plants. Our findings suggest more than one gene confers 2,4-D resistance in KCTR plants. Therefore, it is possible that a combination of P450s or other genes may be involved in metabolic resistance to 2,4-D in KCTR Palmer amaranth. Metabolic resistance to herbicides usually follows a three-step detoxification process, including several genes facilitating herbicide conversion, degradation, and transporting or compartmentalizing of degraded molecules into the vacuole of a plant cell. Metabolic resistance might also involve genes that help in plant recovery after herbicide treatment. More in-depth investigation is needed to decipher the genes involved in the metabolic resistance of 2,4-D in KCTR Palmer amaranth.

## Materials and methods

### Plant materials and growth conditions

2,4-D-resistant (KCTR) and susceptible (KSS; Kansas Susceptible) Palmer amaranth populations were used in this study. KCTR population was originally collected in 2018 from a long-term conservation tilled field with continuous sorghum production in Riley County, Kansas^20^. Although the original field collected seed of KCTR was found resistant to multiple herbicides, including 2,4-D, we selected 2,4-D survivors by treating the plants with 2,4-D at 560 g ae ha^−1^. These plants were kept in isolation under greenhouse conditions to produce next generation of seeds. This process was repeated for two generations so that the seed used in this study were more homogenous in nature with respect to 2,4-D resistance. KSS population is known to be susceptible to 2,4-D and was also collected from a nearby field in Riley County, Kansas and has been characterized previously^20,23,37,38^. Experiments to generate F_1_ and F_2_ progenies for this study were conducted either in greenhouse or controlled environmental growth chambers. The following greenhouse conditions were maintained: 30/24 °C (d/n) temperature and 16/8 h (d/n) photoperiod with natural light supplemented with 250 μmol m^–2^ s^–1^ illumination by sodium vapor lamps. Growth chambers were maintained at 32.5/22.5 °C (d/n) with a photoperiod of 15/9 h (d/n) with 60 ± 10% relative humidity and a light intensity of 750 μmol m^–2^ s^–1^ provided by incandescent and fluorescent bulbs^20^. Seeds of KCTR and KSS Palmer amaranth populations were sown in small plastic trays (21 × 6 × 4 cm) with commercial potting mix (Pro-Mix® premium potting mix, Premier Tech Home and Garden Inc., Ontario, CA) in the greenhouse and allowed to emerge. Palmer amaranth seedlings with 3–4 leaves were transplanted into small pots (6 × 6 × 6.5 cm) for herbicide treatment. Plants were watered as needed (procedure for herbicide treatment is given later).

### Generation of F_1_ and F_2_ families of KCTR Palmer amaranth

**Figure 5:**
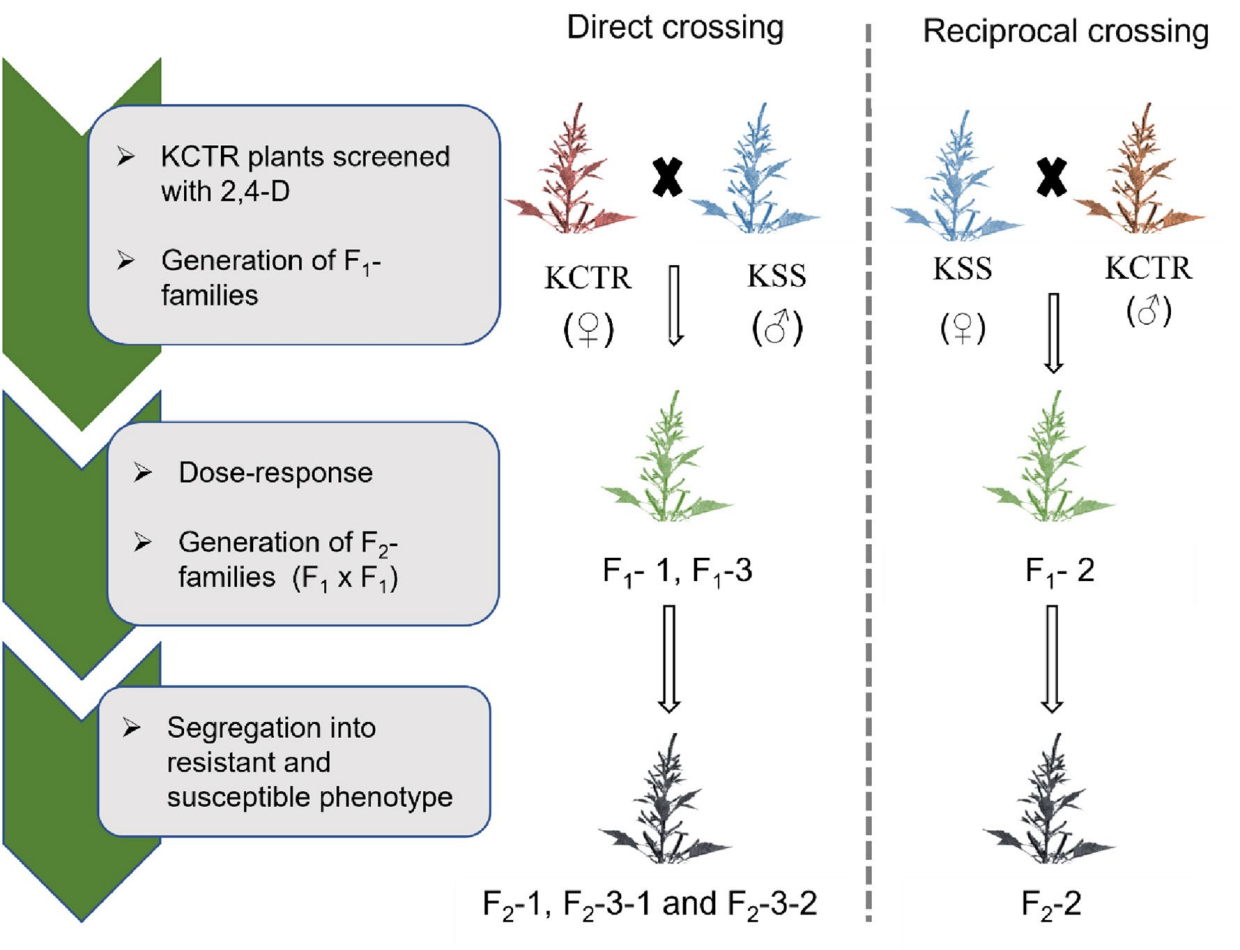
Schematic diagram showing the generation of F_1_ and F_2_ families of 2,4-D susceptible (KSS) and resistant (KCTR) Palmer amaranth and the experimental steps in the study (created with BioRender.com).

Ten-12 cm tall KCTR seedlings (n = 20) were treated when 10–12 cm tall with 2,4-D (2,4-D 4L Amine, Winfield Solutions, LLC., St. Paul, Minnesota, USA) at 1120 g ae ha^−1^ and allowed to grow in the greenhouse. At 4 weeks after treatment (WAT) the survivors were identified and transferred to new pots (15 × 10 × 15 cm) to initiate flowering. Once flowered, healthy male and female KCTR plants were selected for performing direct and reciprocal crosses (Fig. 5). Direct crosses were performed by enclosing female (♀) KCTR (survivors of 2,4-D application) individually with male (♂) KSS (non-treated with 2,4-D) in a pollination bag (Fig. 5). Reciprocal crosses were performed with female (♀) KSS plants (non-treated with 2,4-D) paired with male (♂) KCTR (survivors of 2,4-D application) plants (Fig. 5). Before enclosing the plants, existing flowering parts were removed to ensure crossing between selected plants. Once both plants flowered, male Palmer amaranth plants were gently shaken to disperse pollen and pollinate the stigmas of female plants. At maturity, seeds were collected from each female plant separately, cleaned, and stored at 32 °C in greenhouses to allow over-ripening of the seeds necessary for good germination. In total three F_1_ families were generated from these crosses, *i.e*., F_1_-1, F_1_-2, and F_1_-3 (Fig. 5). Plants from each F_1_ family were further screened with 2,4-D (1120 g ae ha^−1^) to identify female and male F_1_ plants resistant to 2,4-D to be used to generate pseudo-F_2_ families (Fig. 5). In total four F_2_ families were generated from these crosses (F_2_-1, F_2_-2, F_2_-3-1, F_2_-3-2; Fig. 5). These progenies from the F_1_ and F_2_ families were used in further studies.

### 2,4-D dose–response study using KCTR, KSS, and F_1_ progenies

Whole-plant dose–response experiments were conducted with parental KCTR, KSS Palmer amaranth plants, and F_1_ progenies generated from three F_1_ families. The experiments were conducted in a completely randomized design in growth chambers. KCTR, KSS, and progenies from individual F_1_ families were germinated in small trays and transplanted into small pots as described previously. One week after transplanting, the seedlings were transferred to growth chambers (maintained at the same conditions as described in (‘Plant Materials and Growth Conditions’). Palmer amaranth seedlings (10–12 cm tall) were treated with 2,4-D at the following doses i.e., 0 (non-treated), 140, 280, 560 (1×; field recommended dose), 1120, and 2240 (4×) g ae ha^−1^. Thirty minutes after herbicide treatment, plants were returned to the growth chamber. 2,4-D was applied using a bench-track sprayer (Generation III, DeVries Manufacturing, Hollandale, Minnesota, USA) using a flat-fan nozzle tip (8002 Teejet, Spraying Systems Co., Wheaton, Illinois, USA) and was calibrated to spray equivalent to 187 L ha^−1^ (20 GPA). At 4 WAT, plant survival (dead or alive) was assessed at each dose of 2,4-D treatment. Palmer amaranth plants that survived 2,4-D dose (560 g ae ha^−1^ and above) and continued to grow were considered as resistant and those that showed severe epinasty (downward curling of plant parts, a typical symptom of synthetic auxin herbicides) and ceased to grow, were grouped as susceptible to 2,4-D. In response to 2,4-D treatment the plants that survived and Above-ground plant parts were harvested at 4 WAT and oven-dried at 65 °C for at least 72 h. Dose–response experiments with each F_1_ family were repeated once, and 3–5 replications were maintained for each dose of 2,4-D. Dry weight was measured and converted into relative dry weight (% of non-treated) as described below (Eq. 1):

1 $$ {\text{Relative dry weight }}\left( {\% {\text{ of non-treated control}}} \right) \, = \, \frac{{\left( {\text{Dry weight of the sample}} \right) \times { 1}00}}{{ \left( {\text{Average dry weight of non-treated control}} \right)}} \, $$

### Evaluation of segregation (2,4-D resistance or susceptibility) of F_2_ progenies

Seed of F_2_-families (F_2_-1, F_2_-2, F_2_-3-1, F_2_-3-2) were planted and the seedlings were grown in the same greenhouse as described above. Based on the response of parental populations i.e., KCTR and KSS in greenhouse conditions, the field recommended dose of 2,4-D (560 g ae ha^−1^) was identified to discriminate resistant (alive) or susceptible (dead) plants. The same dose was used to determine the segregation of F_2_ progenies into resistant or susceptible plants. About 20–120 progenies (10–12 cm tall; Table 4) representing each F_2_ family were treated with 560 g ae ha^−1^ of 2,4-D. About 20–30 plants of KSS Palmer amaranth were also treated along with the F_2_ progenies as a negative control. Unfortunately, the KCTR plants were not included in this assay due to lack of enough KCTR seed and poor germination at the time of conducting this experiment. At 4 WAT, live or dead (resistance or susceptible) phenotype of the 2,4-D-treated plants was documented (Fig. 5). The experiment was repeated. For the second run of the experiment, at 4 WAT, the above-ground dry weight of F_2_ plants was harvested, dried, and measured (as described above).

### Data analysis

Relative dry weight data from two runs of 2,4-D dose–response experiments of each F_1_ family was compared using Levene's test (α = 0.05) in R (version 4.0.3, R Core Team, 2020) in an R-studio interface. Since it was non-significant, dry weight data were pooled across two runs. Dry weight datasets for each F_1_ family were analyzed using non-linear log-logistic three-parameter ‘*drc*’ models in R^39,40^. The three-parameter regression model used the following formula (Eq. 2):

2 $$ {\text{Y }} = {\text{ d }} + {\text{ exp }}\left[ {{\text{b }}\left( {{\text{log x }}{-}{\text{ log e}}} \right)} \right] $$

where Y is the response variable, x is the herbicide dose, d is the upper limit, b is the relative slope around e and e is GR_50_ (amount of 2,4-D required to reduce plant dry weight by 50%). The GR_50_ values of different F_1_ families and populations were further tested via t-test using the function ‘*compParm*’ in ‘*drc*’ package to determine differences across the doses. In-built ‘*plot*’ function in the ‘*drc*’ package was used to visualize the dose–response curves. Resistance index (RI) was calculated as a ratio of GR_50_ of KCTR or F_1_ families and GR_50_ of KSS population. Cumulative dry weight distribution of the F_1_ progenies along with the parental populations (KSS and KCTR) at each dose of 2,4-D was illustrated (as density plot) using ’*ggplot2*’ package in R^41^. For the plot, dry biomass was represented as bins (each bin represents 0.25 g plant biomass) and proportion of total plants having biomass in that bin was plotted as density.

The number of F_2_ progenies observed to have survived after 2,4-D treatment was compared with the expected number of survivors using chi-square analyses. The expected segregation of 2,4-D resistant (alive) or susceptible (dead) phenotypes was determined based on the Mendelian single locus segregation model, which suggests that the 2,4-D resistance in KCTR is controlled by a single gene. The following Eq. (3) was used to test this assumption.

3 $$ {\text{Exp}}.{\text{ F}}_{{2}} \, = \,({1}\, \times \,{\text{Obs}}.{\text{ KCTR}}\, + \,{2}\, \times \,{\text{Obs}}.{\text{ F}}_{{1}} \, + \,{1}\, \times \,{\text{Obs}}.{\text{ KSS}}) $$

In this equation Exp. F_2_ is the expected segregation frequency of the F_2_ progenies and Obs. is the observed segregation frequency of KCTR, F_1_, and KSS phenotypes. Based on previous experiments conducted in greenhouse conditions, the KCTR plants and F_1_ progenies were found resistant to 560 g ae ha^−1^ of 2,4-D, while KSS plants were susceptible. This dose was used to determine resistant or susceptible plants among F_2_ progenies. Chi-square goodness of fit test (χ^2^) was used to analyze the data of the plant segregation as resistance or susceptible to 2,4-D by comparing the observed segregation frequency with the expected. The null-hypothesis (H_0_) for the test was that segregation of 2,4-D resistance in F_2_ progenies will follow the 3:1 ratio (resistant: susceptible) and p-value from each F_2_ family was compared with the significance level of α = 0.05. The formula used for the test is (Eq. 4):

4 $$ \chi^{{2}} \, = \, \left[ {\left( {{\text{E}} - {\text{O}}} \right)^{{2}} /{\text{ E}}} \right] $$

where χ^2^ is the Chi-square goodness of fit-value, O and E are the observed and expected segregation frequencies, respectively, of progenies in each F_2_ family. H_0_ was rejected if the obtained p-value was < 0.05. Dry weight data of the F_2_ progenies were also visualized as density plot (each bin represents 0.1 g plant biomass).

### Permissions required

All the seed used was collected by the authors from the experimental field maintained by Dr. Peterson, one of the authors of this manuscript.

### Guidelines required

The collection of Palmer amaranth seed used in this study complies with the institutional guidelines.

## Conclusion

Results of this study suggest that 2,4-D resistance in KCTR Palmer amaranth is a nuclear trait, governed by multiple genes. The possible presence of TSR to 2,4-D in KCTR has not been investigated yet and will be interesting future line of work. Future research will also be aimed at discovering metabolic genes that mediate 2,4-D resistance in KCTR. Availability of Palmer amaranth genome^42^ and application of transcriptomic approach can enable identifying specific P450s involved in metabolic resistance in weeds. For example, a P450 gene (*P450-81A10v7*) was documented to confer metabolic resistance to at least 5 SOAs in rigid ryegrass (*Lolium rigidum* L.)^43^. Studies directed to investigate if metabolic resistance to 2,4-D is transferred via pollens to other *Amaranthus *sp. can help in formulating management practices specific to *Amaranthus *sp. Overall, 2,4-D has been a viable option to manage ALS- and EPSPS-inhibitor resistant Palmer amaranth; moreover, 2,4-D resistant crop technology has been shown to provide growers with options to effectively manage glyphosate-resistant Palmer amaranth^44^. However, the evolution of resistance to 2,4-D limits the use of this important herbicide by growers. Therefore, the incorporation of integrated weed management techniques will be necessary for reducing the evolution and spread of 2,4-D resistance.

## Supplementary Information

Supplementary Table S1.

## Data Availability

The datasets used and/or analyzed during the current study available from the corresponding author on reasonable request.
