# Supplementary material for: Inheritance of 2,4-dichlorophenoxyacetic acid (2,4-D) resistance in Amaranthus palmeri
Source: Sci Rep. 2022 Dec 17;12:21822. doi: 10.1038/s41598-022-25686-1 (PMC9759536; doi:10.1038/s41598-022-25686-1)
Supplement: Supplementary file 1 — Supplementary Table S1. [file 41598_2022_25686_MOESM1_ESM.docx]

Table S1. P-values comparing GR_50_ values of KCTR, KSS and F_1_ plants in the F_1_-dose-response analysis.

| Dose-response | Comparison | p-value |
| --- | --- | --- |
| F_1_ | KCTR vs KSS | <0.001 |
|  | KCTR vs F_1_ | 0.14 |
|  | F_1_ vs KSS | <0.001 |
| F_2_ | KCTR vs KSS | 0.016 |
|  | KCTR vs F_2_ | 0.11 |
|  | F_2_ vs KSS | 0.02 |
| F_3_ | KCTR vs KSS | <0.01 |
|  | KCTR vs F_3_ | 0.07 |
|  | F_3_ vs KSS | 0.014 |
